# Trends in inpatient care for psychiatric disorders in NHS hospitals across England, 1998/99–2019/20: an observational time series analysis

**DOI:** 10.1007/s00127-021-02215-5

**Published:** 2021-12-24

**Authors:** Michelle Degli Esposti, Hisham Ziauddeen, Lucy Bowes, Aaron Reeves, Adam M. Chekroud, David K. Humphreys, Tamsin Ford

**Affiliations:** 1 Department of Social Policy and Intervention, University of Oxford, Barnett House, 32 Wellington Square, Oxford, OX1 2ER UK; 2 Department of Psychiatry, University of Cambridge, Cambridge, CB2 3EB UK; 3 Department of Experimental Psychology, University of Oxford, Oxford, OX2 6GG UK; 4 Department of Psychiatry, Yale University School of Medicine, New Haven, CT 06510 USA

**Keywords:** Psychiatry, Mental health, Hospital admissions, Time trends, Hospital episode statistics

## Abstract

**Purpose:**

It is unclear how hospitals are responding to the mental health needs of the population in England, against a backdrop of diminishing resources. We aimed to document patterns in hospital activity by psychiatric disorder and how these have changed over the last 22 years.

**Methods:**

In this observational time series analysis, we used routinely collected data on all NHS hospitals in England from 1998/99 to 2019/20. Trends in hospital admissions and bed days for psychiatric disorders were smoothed using negative binomial regression models with year as the exposure and rates (per 1000 person-years) as the outcome. When linear trends were not appropriate, we fitted segmented negative binomial regression models with one change-point. We stratified by gender and age group [children (0–14 years); adults (15 years +)].

**Results:**

Hospital admission rates and bed days for all psychiatric disorders decreased by 28.4 and 38.3%, respectively. Trends were not uniform across psychiatric disorders or age groups. Admission rates mainly decreased over time, except for anxiety and eating disorders which doubled over the 22-year period, significantly increasing by 2.9% (AAPC = 2.88; 95% CI: 2.61–3.16; *p* < 0.001) and 3.4% (AAPC = 3.44; 95% CI: 3.04–3.85; *p* < 0.001) each year. Inpatient hospital activity among children showed more increasing and pronounced trends than adults, including an increase of 212.9% for depression, despite a 63.8% reduction for adults with depression during the same period.

**Conclusion:**

In the last 22 years, there have been overall reductions in hospital activity for psychiatric disorders. However, some disorders showed pronounced increases, pointing to areas of growing need for inpatient psychiatric care, especially among children.

**Supplementary Information:**

The online version contains supplementary material available at 10.1007/s00127-021-02215-5.

## Introduction

Health services are facing unprecedented financial and operational pressures [1]. With England’s expanding and ageing population, demand for services is outpacing the rate at which the NHS’s budget is growing [2, 3]. A policy objective aimed at meeting this challenge is to develop community-based alternatives to reduce costly inpatient hospital care. Mental health services, in particular, have been transformed to shift the balance away from hospital and towards community care [4]. In the last 30 years, the number of mental health beds has been reduced by 73% [5]. These reductions have released funds in the short term, enabling the locus of psychiatric care to be moved outside the hospital. Although community-based models of care promise early and more sustainable intervention [4], there is now growing evidence that there are too few mental health beds to meet psychiatric need [6–8]. This raises important questions around how shrinking hospital provision impacts on the management of mental health conditions.

With 1 in 4 experiencing a mental health and/or substance use disorder at some point during their lives [9], mental illness has become the single largest cause of disability in England—costing the NHS over £13.3 billion in 2019/20 alone [10]—and is on the rise among both children and adults [11–13]. Repeated population-based surveys find that the prevalence of common mental disorders has increased by 2% from 1999 to 2017 in children and by 2% from 1993 to 2014 in adults [2, 14]. Despite this evidence of deterioration in mental health, it is not possible to draw a direct line from this general rise in poor mental health to an increased need for acute psychiatric care as only 0.2% of people with poor mental health and 1.8% of people with severe mental health disorders receive inpatient care among adults [2]. Inpatient admission for children’s mental health is even less common.

**Fig. 1:**
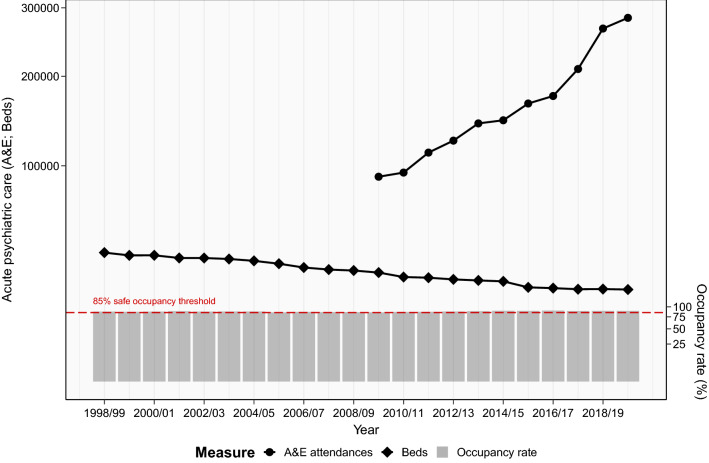
Measures of acute psychiatric provision from 1998/99 to 2019/20, including counts of A&E attendances for psychiatric conditions (circle scatter plot), mental health beds (diamond scatter plot), and rate of mental health bed occupancy (%, grey bar chart). Where A&E attendances and occupancy rates indicate need, while beds represent resources. Y-axes are plotted on a square-root scale

However, measures of hospital activity for mental health point towards an increasing need for acute psychiatric care, despite diminishing resources. Since 1998/99 occupancy rates of mental health beds have exceeded the recommended 85% threshold for delivering high quality and safe care [8, 15]. Occupancy rates have risen even further in the last 10 years, resulting in patients having to be admitted out-of-area, often at considerable distance from home, because of a lack of local beds [16, 17]. In addition, A&E activity for psychiatric conditions more than doubled between 2009/10 and 2019/20, further suggesting an increasing need for acute psychiatric care [18]. Taken together, this suggests a divergence between need and provision as hospital activity for psychiatric conditions increases, while resources for inpatient care are reduced (see Fig. 1).

Those receiving inpatient care seem to be more unwell. Based on scores on the Health of the Nation Outcome Scales (HoNOS), a widely used measure of health, impairment, and social functioning in adults with mental illness, adults admitted to inpatient care in 2018 were found to be more severely unwell (higher average HoNOS scores), than those admitted in 2013. Similarly, at discharge, average HoNOS scores at the point of discharge have been higher in recent years [8]. These comparisons indicate that the threshold for admitting people with severe mental illness has increased, while thresholds for discharging patients have fallen. In addition, compulsory detentions under the Mental Health Act have more than doubled over the last 30 years [19, 20]. It is unclear to what extent this is because improved community services are able to look after people with severe mental illness in the community with only the most severely ill needing hospital admission, or because the shortage of mental health beds means they have to be prioritised for the most unwell [20–22]. Nevertheless, the fact that compulsory detentions make up a higher proportion of mental health bed occupancy reflects the severity of illness in those receiving hospital care.

While reductions in hospital provision are well documented, it is less clear how the remaining resources are being allocated in a system under strain. In this study, we use Hospital Episode Statistics (HES), which are routinely collected data from all hospitals across England [23], to explore how inpatient care (hospital admissions and bed days) is being used to meet acute psychiatric need, and if and how this has changed over the last 22 years as need increases and hospital provision continues to diminish.

## Method

This observational time series analysis used routinely collected data on inpatient hospital activity in England from 1998/99 to 2019/20. Ethical approval was not required, because all data were fully anonymised, pre-published, and publicly available. In line with best-reporting practices, we followed general recommendations set-out in the REporting of studies Conducted using Observational Routinely collected health Data (RECORD) statement [24].

## Data source

The Department of Health routinely collects data on all admissions to NHS hospitals in England and independent sector providers (private or charitable hospitals) commissioned by the NHS: Hospital Episode Statistics Admitted Patient Care (HES APC) [23]. HES APC offers the opportunity to estimate population-based hospital admissions for psychiatric disorders as it measures all NHS hospital admissions. A hospital admission covers any secondary care-based activity that requires a hospital bed, including both emergency and planned admissions and day cases, but not including accident and emergency (A&E, emergency department) attendances, or outpatient bookings. HES data, therefore, include hospital admissions that have been made following contact with specialist mental health services, as well as admissions after attending A&E. We extracted the primary diagnoses for each HES APC entry, which represents the main reason the patient is receiving hospital care. Diagnoses were coded using the International Classification of Diseases version 10 (ICD-10). The HES APC database is collated and curated by NHS Digital.

## Study data

We obtained HES data for each financial year (1st April to 31st March) from 1998/99 to 2019/20. These dates were chosen as it was the longest available time period with consistent ICD codes (i.e., version ICD-10). We used ICD-10 codes for common mental, behavioural, and neurodevelopmental disorders (see Supplementary Table 1 for full details) for primary diagnoses (3-character) to consistently identify hospital admissions and bed days over time. Outcome measures were aggregated for all common psychiatric disorders (ICD-10 codes: F00- F91) and broken down by specific disorder type (e.g., depression, see Supplementary Table 1).

## Main outcomes

Our main outcomes were hospital admissions and overnight bed use, measured by bed days and length of stay. For hospital admissions and episodes, we modelled age-specific rates per 1000 person-years to control for changes in population size and by age group. We obtained age-specific population estimates for England from the Office for National Statistics’ (ONS) mid-year population estimates.

### Admissions

Rates of admissions (or finished admission episodes) per 1000 person-years of the total population. Hospital admissions represent the first period of inpatient care under one consultant within one healthcare provider. Admissions are counted against the year in which they end, and do not represent the number of patients, as a person may have more than one admission within the year [23]. Because admissions measure the first period of care, they are less prone to double counting from changes to the provision of inpatient care. As a result, they represent a subset of all hospital episodes—around 5% of admissions have more than one episode of inpatient care.

### Bed days and length of stay

Total bed days represent the sum of hospital episode duration, from admission to discharge date, for all episodes ending in the year. Measures of total bed days changed over the study period: from 1998/99 to 2007/08, bed days included estimates of unfinished episodes where finished episodes were uplifted by a factor to capture episodes that were not finished at the end of the year, whereas from 2008/09 onwards, total bed days only captured finished episodes for that year and were not uplifted by any estimation factor. Because any observed changes in bed days from 2008/09 may be confounded by this measurement change, we supplemented bed days with measures of length of stay. Information on length of stay was only available as summary statistics (mean or median) aggregated for each diagnosis for each year. To guard against skew from outliers, we extracted the median length of hospital spell duration in days instead of the mean. A spell is a period of continuous admitted patient care within a particular provider, calculated by subtracting the admission date from the discharge date for finished episodes. Day cases, which have a length of stay of zero bed days, were excluded from both estimates of total bed days and median length of stay.

## Secondary outcomes

To characterise patients being admitted for psychiatric hospital care, we obtained information on finished consultant episodes broken down by gender and age.

### Episodes by gender and age

Rates of episodes (or finished admission episodes) per 1000 person-years of the age-specific population. Finished consultant episodes (FCE) represent a continuous period of admitted patient care under one consultant within one healthcare provider. FCEs are counted against the year in which they end and are more prone to double counting as a person may have more than one episode of care within the same stay in hospital or in different stays in the same year [23]. Admissions represent a subset of FCEs as they measure only the first FCE and are therefore less susceptible to double counting (see Supplementary Fig. 1). Unlike admissions however, information on gender and age were publicly available for FCEs for our study period. We extracted demographic information on gender (male, female, and unspecified gender) and age (0–14y; 15y+). These gender and age categories were used as only information on males (i.e., not on females) and broader age groups (age 0–14y; 15–59y; 60–74y; 75y+) were collected consistently over time.

## Statistical analysis

We analysed trends in the yearly hospital admissions for psychiatric disorders by fitting negative binomial regression models with year as the exposure and the number of admissions as the main outcome, as there was significant evidence (*p* < 0.05) of overdispersion [25]. Year and age-specific population estimates (log-transformed) were included as an offset variable to adjust for changing population size and age structure. To handle any non-linearity between year and psychiatric hospital episodes, and more accurately model rate of change over the study period, we conducted change-point analyses. Change-point analysis is a data-driven, exploratory method that checks whether the outcomes follow a consistent linear trend over time or may be better explained by two trends—allowing for one change in slope gradient and/or trend direction [26]. For our change-point analyses, we compared simple linear (continuous) models against segmented regression models with two slopes, including a parameter for the time at which the change of slopes occurred (i.e., the change-point). Change-points were estimated by iteratively fitting the linear model with a linear predictor through the *segmented* package in R (version 3.5.2) [27]. We selected the best-fitting model based on Akaike’s information criterion (AIC)—low AIC values were preferred—and performing the (pseudo) score statistics to evaluate whether incorporating a change-point improved model fit (*p* < 0.05) [28].

We examined both annual percentage and overall changes to interpret trends. We calculated the average annual percentage changes (AAPCs) and *p* values from our best-fitting regression models (i.e., simple or segmented, see above). AAPCs represent whether trends linearly increase or decrease from 1998/99 to 2019/20 while accounting for the uncertainty in the detected change points for segmented models [28, 29]. APPCs further estimate the average amount of percentage change for each year and indicate whether these year-on-year changes are significant. We supplemented these measures by calculating overall absolute change and relative change (i.e., percentage change) for the earliest year (1998/99) and most recent (2019/20) available year, as well for the estimated change-point and most recent year (2019/20) for segmented models. To visualise trends, we plotted estimated counts from the best-fitting models (simple or segmented) and thus most accurately modelled changes over time.

## Results

Table 1 describes inpatient care for psychiatric disorders from 1998/99 to 2019/20. Alcohol use disorder resulted in the highest number of hospital admissions and episodes (Supplementary Fig. 1), accounting for 26.9% of adult and 47.6% of child inpatient episodes. However, alcohol use disorder only accounted for 4.5% of bed days as it had the second shortest hospital stay. Schizophrenia had the second highest number of admissions and accounted for almost half of all bed days (42.7%), with the length of hospital stay only exceeded by dementia and bipolar disorder (Supplementary Fig. 2). Eating disorders (17.8%) and anxiety disorders (10.8%) were the next most common indication for children’s inpatient care. There was little difference between males and females across all disorders (Supplementary Fig. 3), though expected differences were seen within disorders (Table 1 & Supplementary Fig. 4). For example, alcohol and substance use disorders were more common among males (70–72%), whereas personality and eating disorders were more common among females (67–91%). The overall percentage of males admitted to hospital increased from approximately 50% in 1998/99 to 55% in 2019/20 (see Supplementary Fig. 3).

**Table 1:** Descriptive statistics for hospital admissions and bed days for psychiatric disorders, 1998/99–2019/20

	Hospital admissions (count)	Total bed days (count)	Median length of stay (mean (SD))	FCEs				
				Total (count)	Gender (all ages)		Age (all genders)	
					Male (%)	Female & unknown (%)	Child (%)	Adult (%)
Common psychiatric disorders^**‡**^	3,333,418	123,531,860	14.8 (1.66)	4,267,571	53.65	46.35	1.5	98.08
Alcohol use disorder	865,942	5,522,892	2.23 (1.34)	1,160,429	69.96	30.04	2.62	96.84
Anxiety	184,249	2,401,566	5.6 (3.26)	214,091	36.46	63.54	3.21	96.52
Bipolar disorder	231,975	12,181,797	30.68 (2.32)	289,348	40.99	59.01	0.11	99.53
Conduct disorders	7037	116,259	1.91 (1.02)	7651	60.68	39.32	50.86	48.88^†^
Dementia	359,665	19,012,144	33.14 (7.07)	551,242	41.68	58.32	0.01	99.78
Depression	506,585	18,038,321	20.39 (3.07)	580,579	40.31	59.69	0.77	98.95
Eating disorders	48,564	2,106,319	15.00 (3.77)	62,840	9.30	90.70	18.04	81.73
OCD	12,756	626,580	20.43 (2.75)	14,305	52.51	47.49	6.81	92.94
Personality disorders	190,238	6,198,083	10.36 (1.11)	224,492	32.39	67.61	0.13	99.33
PTSD	109,437	1,931,199	7.82 (0.66)	122,050	52.14	47.86	1.08	98.52
Schizophrenia	642,799	52,738,338	25.26 (2.49)	839,449	61.8	38.20	0.25	99.35
Substance use disorders	174,171	2,658,363	4.75 (1.78)	201,095	71.73	28.27	0.94	98.01
Population estimates (per million)	1148.66	–	–	1148.66	564.19	584.47	208.52	940.14

*FCEs* finished consultant episodes,* OCD*  obsessive–compulsive disorder, *PTSD* post-traumatic stress disorder, *SD* standard deviation

Child includes individuals aged 0–14 years and adult includes individuals ages 15 years and older; percentages for age groups do not add up to 100% due to missing information

**‡**Represents the summed total of the 12 most common psychiatric disorders (see Supplementary Table 1 for full diagnostic details)

^†^Because adults represent ages 15y + , conduct disorders in this age group reflect conduct disorders among adolescents

**Fig. 2:**
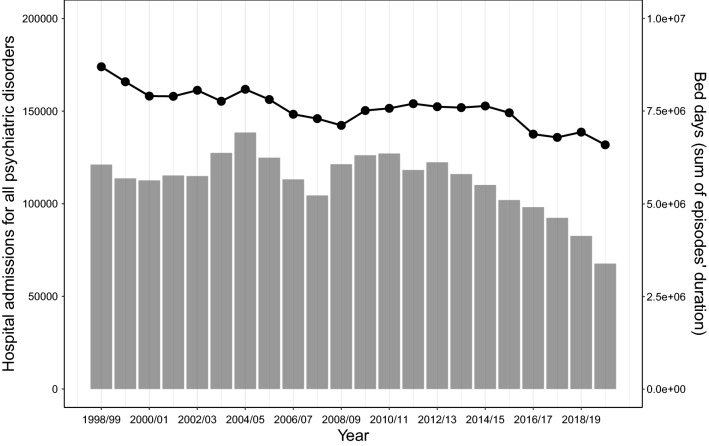
Hospital admissions and bed days for all 12 common psychiatric disorders from 1998/99 to 2019/20

Three-year moving averages identified that the number of hospital admissions for all psychiatric disorders decreased by over 18.1% across the 22-year period (see Fig. 2). The equivalent of around 5000 fewer yearly hospital admissions for psychiatric disorders, even though England’s population size increased by around 13.8% during this period (Supplementary Table 2). Modelling trends in admission rates while adjusting for changing population size identified a larger overall reduction of 28.4% (Table 2). Bed days also decreased over this period, with a reduction of 26.4% from 1998/99 to 2019/20 (Fig. 2 & Supplementary Table 3). A sensitivity check using median length of stay mirrored the same decreasing trend as total bed days (Supplementary Fig. 5).

**Table 2:** Trends in yearly hospital admission rates and bed days for psychiatric disorders, 1998/99–2019/20

	Best-fitting model	Year of change^†^	AAPC (95% CI)	Estimated rates/counts			Overall change from 1998/88 to 2019/20		Change from year of change to 2019/20	
				1998/99	Year of change	2019/20	Absolute change	Relative change	Absolute change	Relative change
**Hospital admissions (rates per 1000 person-years)**										
Common psychiatric disorders^‡^	Simple	–	− 1.58 (− 1.8–− 1.35)^***^	3.43	–	2.45	− 0.97	− 28.4%	–	–
Alcohol use disorder	Segmented	2010/11	1.34 (1.14–1.53)^***^	0.55	0.90	0.72	0.18	32.1%	− 0.18	− 20%
Anxiety	Segmented	2000/01	2.88 (2.61–3.16)^***^	0.12	0.12	0.22	0.10	81.6%	0.11	91.1%
Bipolar disorder	Segmented	2015/16	− 3.64 (− 3.85–− 3.42)^***^	0.26	0.18	0.12	− 0.14	− 54.1%	− 0.06	− 33.3%
Conduct disorders	Segmented	2003/04	− 7.09 (− 7.44–− 6.73)^***^	0.02	0.01	0.00	− 0.01	− 78.6%	0.00	− 41.6%
Dementia	Segmented	2009/10	− 5.55 (− 5.82–− 5.29)^***^	0.58	0.27	0.17	− 0.40	− 69.9%	− 0.10	− 36.3%
Depression	Simple	–	− 5.55 (− 5.77–− 5.33)^***^	0.76	–	0.23	− 0.53	− 69.8%	–	–
Eating disorders	Segmented	2003/04	3.44 (3.04–3.85)^***^	0.03	0.03	0.06	0.03	103.5%	0.03	100.5%
OCD	Simple	–	− 3.46 (− 3.88–− 3.05)^***^	0.02	–	0.01	− 0.01	− 52.3%	–	–
Personality disorders	Segmented	2006/07	0.61 (0.10–1.12)^*^	0.18	0.14	0.20	0.02	13.6%	0.06	44.8%
PTSD	Simple	–	− 2.18 (− 2.88–− 1.47)^***^	0.12	–	0.08	− 0.04	− 37.0%	–	–
Schizophrenia	Simple	–	− 2.10 (− 2.54–− 1.65)^***^	0.70	–	0.45	− 0.25	− 35.9%	–	–
Substance use disorders	Segmented	2008/09	− 0.80 (− 1.13–− 0.47)^***^	0.19	0.13	0.16	− 0.03	− 15.5%	0.04	28.4%
**Bed days (summed counts)**										
Common psychiatric disorders^‡^	Segmented	2013/14	− 2.27 (− 2.54–− 2.01)^***^	5,915,432	6,069,844	3,649,095	− 2,266,338	− 38.3%	− 2,420,749	− 39.9%
Alcohol use disorder	Segmented	2011/12	− 2.49 (− 2.7–− 2.27)^***^	286,130	262,624	168,585	− 117,545	− 41.1%	− 94,039	− 35.8%
Anxiety	Segmented	2012/13	− 1.66 (− 2.03–− 1.30)^***^	119,418	111,765	84,061	− 35,357	− 29.6%	− 27,704	− 24.8%
Bipolar disorder	Segmented	2013/14	− 2.55 (− 2.83–− 2.27)^***^	590,970	584,339	343,404	− 247,566	− 41.9%	− 240,935	− 41.2%
Conduct disorders	Simple	–	− 8.90 (− 10.66–− 7.10)^***^	11,930	–	1685	− 10,245	− 85.9%	–	–
Dementia	Segmented	2012/13	− 5.82 (− 6.09–− 5.54)^***^	1,246,951	801,333	354,204	− 892,747	− 71.6%	− 447,129	− 55.8%
Depression	Segmented	2004/05	− 5.29 (− 5.52–− 5.05)^***^	1,241,499	1,081,380	396,862	− 844,637	− 68.0%	− 684,518	− 63.3%
Eating disorders	Segmented	2015/16	3.08 (2.51–3.66)^***^	59,610	128,841	112,783	53,173	89.2%	− 16,058	− 12.5%
OCD	Simple	–	− 0.93 (− 1.74–− 0.12)^*^	31,379	–	25,761	− 5617	− 17.9%	–	–
Personality disorders	Simple	–	3.00 (2.14–3.87)^***^	203,001	–	377,905	174,904	86.2%	–	–
PTSD	Simple	–	− 1.33 (− 1.93–− 0.73)^***^	100,668	–	76,001	− 24,667	− 24.5%	–	–
Schizophrenia	Segmented	2013/14	− 0.36 (− 0.72–− 0.01)^*^	1,882,476	3,033,112	1,743,817	− 138,659	− 7.4%	− 1,289,295	− 42.5%
Substance use disorders	Simple	–	− 1.16 (− 1.74–− 0.57)^***^	136,138	–	106,665	− 29,472	− 21.7%	–	–

†Year of change is given only if the change-point (or segmented) model detected a significant deviation from a linear trend based on the pscore test

For change-point models, AAPC represents the average annual percentage change from 1998/99 to 2019/20 while accounting for the uncertainty in the detected change points [28, 29]

‡Represents the summed total of the 12 most common psychiatric disorders (see Supplementary Table 1 for full diagnostic details)

*p < 0.05; **p < 0.01; ***p < 0.001

### Trends in hospital admissions by psychiatric disorders

**Fig. 3:**
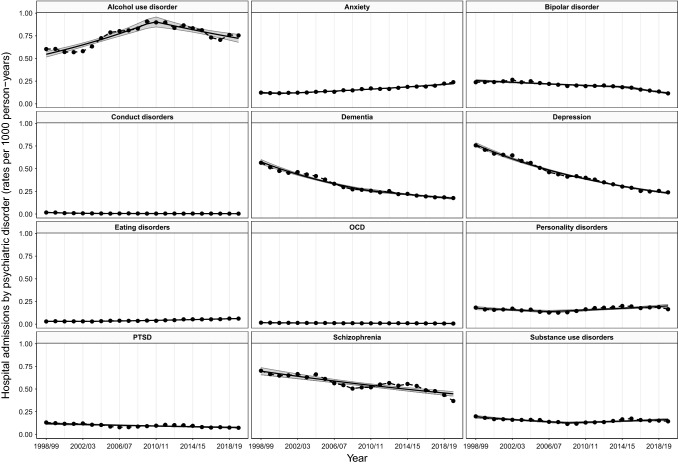
Trends in hospital admission rates by psychiatric disorder, 1998/99–2019/20

Trends in rates of hospital admissions varied by psychiatric disorder (Table 2; Fig. 3). Admission rates for seven out of the 12 psychiatric disorders showed overall reductions from 1998/99 to 2019/20: bipolar disorder, conduct disorders, dementia, depression, OCD, PTSD, and schizophrenia. Although the magnitude of the rate of change was not consistent over time for bipolar disorder, conduct disorders and dementia, all seven disorders significantly decreased each year. The largest reductions of around 70% were seen for conduct disorders, dementia, and depression—equivalent to a year-on-year decrease of 5.6–7.1%—while schizophrenia showed the smallest significant decrease of 35.9%. Only two psychiatric disorders, anxiety and eating disorders, showed consistently increasing trends, with admission rates almost doubling between 1998/99 and 2019/20, translating to relative increases of 81.6 and 100.5% (respectively). These temporal patterns result in anxiety and eating disorders accounting for a larger proportion of all common psychiatric admissions in 2019/20 compared to 1998/99 (Supplementary Fig. 6).

Trends admission rates for three psychiatric disorders changed direction during the study period (Fig. 3). Admission rates for alcohol use disorder sharply increased from 1998/99 to 2010/11 and then shallowly decreased from 2010/11 onwards. Since the initial period of increase was greater than the following period of decrease, this resulted in an overall 32.1% increase. Substance use and personality disorders showed the opposite pattern: initially decreasing and then increasing. These changing trends meant that the average annual percentage changes for these disorders were small, ranging from − 0.8–0.6%. However, when comparing from their estimated change points of 2008/09 for substance use disorders and 2006/07 for personality disorders, both showed notable increases of 28.4 and 44.8%, respectively.

### Trends in hospital bed days by psychiatric disorders

**Fig. 4:**
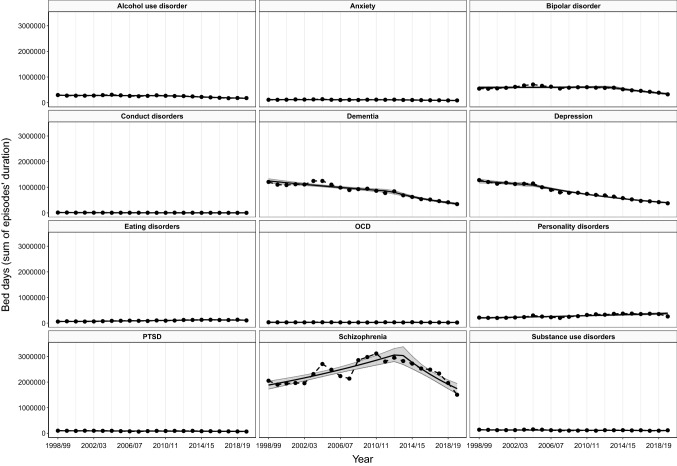
Trends in hospital bed days (total counts) by psychiatric disorder, 1998/99–2019/20

Trends in the total number of hospital bed days typically mirrored trends in admissions (see Fig. 4). Bed days for bipolar disorder, conduct disorders, dementia, depression, OCD, and PTSD all declined at a similar yearly rate to admissions. For example, from 1998/99 to 2019/20, admission rates for depression significantly decreased by 5.5% each year (AAPC: − 5.55; 95% CIs: − 5.77 to − 5.33; *p* < 0.001), while bed days also decreased by a similar magnitude of 5.3% each year (AAPC: − 5.29; 95% CIs: − 5.52 to − 5.05; *p* < 0.001). However, a diverging pattern was seen with some disorders. Despite a substantial 81.6% rise in rates of admissions for anxiety, bed days reduced by 29.6% over the same period. While admissions for eating disorders followed a steep and consistent rate of increase from 2003/04 onwards, bed days for eating disorders went from increasing to decreasing by 12.5% from 2015/16 to 2019/20. Admission rates for alcohol use disorder increased by 32.1% over the study period and by 28.4% for substance use disorders from 2008/09, but bed days for both conditions consistently decreased.

These discrepancies in the total number of bed days compared to admissions may be explained by changes in median length of hospital stay for each admission. Although median length of hospital stay was relatively stable over time, shortening of hospital stays was visible for alcohol use disorder, anxiety, conduct disorders, eating disorders, and substance use disorders (see Supplementary Fig. 7). For example, length of stay for anxiety reduced from 10.3 days down to 2.1, shortening the typical length of hospital by around 80% (see Supplementary Table 4).

### Trends in psychiatric hospital episodes by age group

Rates of hospital episodes for psychiatric disorders disaggregated by adults and children are presented in Supplementary Table 5. Trends in rates for adults followed the same patterns as overall admission rates (Supplementary Fig. 8), since most hospital episodes were for adults (Table 2). However, trends for children showed marked differences, where there were pronounced increases in mental health disorders requiring hospital care compared to adults (Supplementary Fig. 9). For example, despite hospital activity for depression consistently declining by 4.7% each year among adults (AAPC: − 4.72; 95% CIs: − 5.00 to − 4.43; *p* < 0.001), it consistently increased by 5.6% among children (AAPC: 5.58; 95% CIs: 4.48–6.69; *p* < 0.001). This meant that trends in depression showed stark diverging trends by age group, with large reductions in adults yet large upsurges in children. Although hospital care for anxiety and eating disorders increased for both age groups, the rise was more pronounced among children. Anxiety increased by 283.3% and eating disorders by 361.2% in children, compared to 98.1 and 167.6% (respectively) in adults. In addition, there was evidence of more hospital activity over time for OCD, PTSD, personality disorders, and schizophrenia for children compared to adults (see Supplementary Table 5).

## Discussion

Using national data on hospital usage, we found that over the period 1998/99 to 2019/20, admissions for psychiatric disorders decreased by around 28% and the total number of bed days decreased by 38%. These reductions in psychiatric hospital care were not uniform across mental health disorders or across age groups. Alcohol use disorder was the largest single reason for being admitted to hospitals for both children and adults, followed by schizophrenia and depression in adults, and by eating disorders and anxiety in children. For adults, admissions for most psychiatric disorders declined consistently, but those for anxiety and eating disorders doubled from 1998/99 to 2019/20. For children, on the other hand, admissions increased for several disorders. These changes in admissions over time were not always mirrored by changes to total number of bed days, reflecting the fact that the length of hospital stay for some disorders has been dramatically shortened in recent years. Our findings show that reductions in psychiatry hospital activity over the last 22 years have not occurred for all disorders or for children, highlighting areas of growing need for acute psychiatric care.

These findings are against the backdrop of long-term reductions in hospital provision and known shortages in mental health beds [5], as well as reductions in the resourcing of community mental health and social care during the study period. The HONOS data show that over time people are more unwell at admission and at discharge, pointing towards a growing pressure on inpatient resources. The known shortage in inpatient beds has previously been linked to subsequent increases in involuntary psychiatric hospital admissions, as the discrepancy between demand and supply may, in and of itself, exacerbate subsequent demand [21]. Cuts to community mental health and social care services that may have previously curtailed such demand for inpatient care by preventing hospital admissions could be further contributing to these changes [30]. An increased emphasis on community mental health provision through home treatment teams and assertive community outreach (ACT) may have helped to reduce inpatient care for severe mental illness since 2000 [31, 32]. However, it is important to bear in mind that home treatment teams were an integral part of bed reduction initiatives rather than a factor that led to a reduced need for beds, and many ACT teams across England have been decommissioned and disbanded in the last 10 years, leaving a potential demand for acute psychiatric care which hospitals are now under resourced to provide for.

A growing body of evidence shows that acute psychiatric care is a system under strain. The different trends in hospital admissions for psychiatric disorders may therefore reflect practical and operational decisions around service allocation. Patients with specific psychiatric disorders can be more easily allocated to general and acute hospital beds or specialist beds for specific diagnoses. For example, approximately 20% of hospital admissions are for patients with dementia—many of which go on to occupy general and acute hospital beds [33]. While the number of general and acute hospital beds has also reduced over time, these reductions have been less severe compared to the reductions in mental health beds [5]. In addition, multidisciplinary specialist inpatient dementia units (SIDU) have been developed within acute trusts to provide hospital care for dementia patients with more complex needs [34]. As a result, patients with psychiatric disorders that can be allocated to general acute hospital beds and dedicated specialist units, such as dementia, may be subject to less downward pressure than those requiring mental health beds specifically. The provision of inpatient psychiatric care for children and adolescents may also be subject to unique operational pressures. This is supported by figures showing an increasing number of children and adolescents being inappropriately placed in adult psychiatric wards, as well as inappropriate out-of-area placements, to receive acute psychiatric care [35, 36]. Again, however, the burden of supplying psychiatric beds to children and adolescents in need will vary by psychiatric disorders, since patients with some mental health problems (e.g., eating disorders) can more readily receive acute care in general paediatric wards compared to other mental health problems (e.g., schizophrenia) [37].

The changing trends in hospital admissions do not closely match changes in prevalence of psychiatric disorders. Although we identified increases in inpatient care for mood-related disorders which may reflect increases in their prevalence in the general population, the changes in hospital admissions exceed the documented rises in disorder prevalence in the population [2, 38]. In the case of anxiety disorders, rates of hospital admissions almost doubled from 1998/99 to 2019/20, while the prevalence of anxiety as identified by population surveys from 1997 to 2017 only increased by 2% [2]. This suggests that other factors are contributing to these increases in admissions for anxiety and personality disorders, including a lack of—or at least reduction in—community services for these conditions. On the other hand, the 70% reduction in hospital admissions for both depression and dementia is unlikely to reflect a decrease in the population prevalence of these disorders in England, given the evidence from national surveys and projections from modelling studies [2, 39]. This suggests that acute psychiatric services may not be providing for all people living with these psychiatric disorders, either because these people are receiving adequate care elsewhere (e.g., in the community) or because these psychiatric disorders are considered less of a priority for inpatient care when there are bed shortages.

Wider societal and economic factors need to be considered as well. Public health initiatives, such as those restricting the availability, affordability, and marketing of alcohol, may be contributing to a decline in drinking, even at the extreme end of the spectrum [40]. However, it is possible that reductions in hospital admissions for alcohol use disorder reflect changes in the frequency and patterns of substance use in the population with other substances becoming more prominent, which may explain the increase in admission for substance use disorder at a similar to time to the decline in admissions for alcohol use disorder. Another important factor here may be the impact of the economic recession in 2008/10 on inpatient psychiatric activity [41]. The significant cuts to mental health and social care following the recession, coupled with heightened financial and job instability, may have contributed to and worsened poor mental health [42]. Such findings need further careful examination to establish the effects of economic downturns on rates of psychiatric hospital activity [43]. Given the COVID-19 pandemic and the pressing need for services to effectively anticipate and respond to changing demands [11, 44], understanding the relationship between economic instability and community and inpatient psychiatric activity at the national level should be a research priority.

Our results identified more increasing and pronounced trends in psychiatric hospital care for children [45]. The change in the population prevalence of these conditions is similar among both adults and children and so these trends suggest that children are disproportionately requiring inpatient psychiatric care. CAMHS services turn away 1 in 4 referrals (around 52,500 children each year) [46]. Untreated, these patients may later present in crisis and place additional demands on the acute hospital setting. Inpatient hospital activity for children with eating disorders showed particularly steep increases [31], despite evidence showing that hospital care is not the best first line option [47]. Specifically, research indicates that family treatment outside of the hospital setting and specialist community services may be more effective than general child and adolescent mental health services (CAMHS) for treating young people with eating disorders [48]. Admissions for depression and schizophrenia were also rising faster among children than adults. Both trends suggest a need for improved prevention programmes, lower acuity treatment options, and community care to reduce the demand for hospital care through effective early intervention for children specifically. Future research should focus on improving our understanding of the role of specialist community services in preventing costly admissions to hospitals.

## Study limitations

This was a descriptive association study and did not formally examine the role of causal factors (e.g., 2008/10 economic recession) in driving trends and changes over time. This paper cannot speak to why we observed these temporal patterns in hospital admissions for psychiatric disorders. Instead, our modelling approach aimed to descriptively summarise trends over the last 22 years by fitting linear or segmented linear models with one change-point. As a result, the models for some psychiatric diagnoses showed superior fit to others. Notably, the simple linear models failed to capture substantial variation in rates of hospital admissions for PTSD and schizophrenia, which both dipped around 2006/07 to 2008/09 and then spiked between 2011/12 and 2014/15. HES routine data are an invaluable source, but routinely collected data have their limitations. The data used in this study are restricted to admitted patient care and will thus underestimate the true burden on hospitals as they do not capture all A&E or day attendances. We extracted and restricted the analyses to psychiatric disorders which were coded as the primary diagnosis—the main reason for receiving hospital care—to minimise double counting from secondary diagnoses and avoid confounding from changing recording practices around dual diagnoses. Although this makes for a more focused and cleaner time trends analysis, it does miss out on important information on potential co-morbidities. In addition, the disaggregated data were only available for the age groupings (0–14y and 15y+) over the study period, which do not map on to NICE-recommended divisions and obscure information on adolescents. Unlike gender and age which are well completed, we were unable to analyse data on ethnicity due to inconsistent and incomplete recoding [23]. We were also unable to analyse differences in hospital providers (general vs psychiatric hospitals) or type of psychiatric admission (voluntary vs involuntary) as these data are not published in the HES APC database. Up-to-date statistics on inpatients detained under the Mental Health Act (1983) are not yet available (last reporting period 2015/16). Patterns in involuntary psychiatric admissions detained under the Mental Health Act may play a key role in driving overall trends as previous evidence shows significant rises in involuntary admissions since the 1980s, which has been linked to economic deprivation, and warrants further investigation [19, 21, 49]. Finally, changing recording practices and priorities may undermine the temporal consistency of the psychiatric diagnoses. For example, in January 2007, there was a move from secondary uses clinical information being stored and managed by the NHS-Wide Clearing Service (NWCS) to the Service Secondary Uses Submissions (SUS), with the aim to make more clinical information electronically available. There were also changes to how the total number of bed days were estimated in 2003 and 2008. Although this may have affected the temporal consistency of the data during our study period, we did not identify unanimous changes in any of these years across psychiatric disorders. This suggests that the patterns seen for psychiatric hospital activity cannot be fully attributed to these recording changes and our smoothing of the trends are likely to have (at least partially) controlled for such inconsistencies over time.

## Conclusions

Admissions to hospitals are expensive, and current policy objectives and guidance focus on trying to maintain support for individuals with poor mental health outside of the hospital setting. However, the patterns of hospital admissions identified here, and how they have changed over the last 22 years, show that not all psychiatric disorders or age groups are experiencing reductions in hospital activity. We observed increases in hospital admissions for anxiety and eating disorders, especially among children. This raises critical questions around whether the large-scale reductions in hospital provision and mental health beds over the last 30 years, alongside the reductions in community mental services in recent years, have left people more vulnerable and more likely to present to hospitals in crisis—even though hospitals have diminishing resources to provide for these acute mental health needs.

## Supplementary Information

Below is the link to the electronic supplementary material.

Supplementary file1 (PDF 118 KB)

Supplementary file2 (PDF 740 KB)

## Data Availability

All data are freely and publicly available from NHS Digital. A dataset of aggregated yearly data on Admitted Patient Care (Hospital Episode Statistics) is also available on request from the corresponding author at mdesposti@gmail.com.

## Code availability

All statistical code used in the analyses has been deposited at the Open Science Framework (https://osf.io/gn65w/?view_only=4b3cdfc9787d40d0bcf9a53faa36471c) for purposes of reproducing or extending the analysis.
