# Supplementary material for: Trends in inpatient care for psychiatric disorders in NHS hospitals across England, 1998/99–2019/20: an observational time series analysis
Source: Soc Psychiatry Psychiatr Epidemiol. 2021 Dec 24;57(5):993–1006. doi: 10.1007/s00127-021-02215-5 (PMC8705084; doi:10.1007/s00127-021-02215-5)
Supplement: Supplementary file 2 — Supplementary file2 (PDF 740 KB) [file 127_2021_2215_MOESM2_ESM.pdf]

Trends in inpatient care for psychiatric disorders in NHS hospitals across England, 1998/99-2019/20: an observational time series analysis

Supplementary Material

Contents **Page**

Supplementary Tables

Supplementary Table 1. ICD-10 codes for common mental, behavioural and neurodevelopmental disorders 2

Supplementary Table 2. Three year moving averages for the total counts of hospital admissions by psychiatric disorder and population estimates, 1998/99-2019/20 3

Supplementary Table 3. Three year moving averages for the total counts of bed days by psychiatric disorder, 1998/99-2019/20 4

Supplementary Table 4. Three year moving averages for the median length of stay in hospital by psychiatric disorder, 1998/99-2019/20 5

Supplementary Table 5. Trends in yearly hospital episode rates by age group (adults and children), 1998/99-2019/20 6

Supplementary Figures

Supplementary Figure 1. Hospital activity for psychiatric disorders by measure (admission *vs* finished consultant episode). Averaged across the full study period (1998/99-2019/2020). 7

Supplementary Figure 2. Median length of hospital stay by psychiatric disorder. Averaged across the full study period (1998/99-2019/2020), with error bars representing standard deviations. 8

Supplementary Figure 3. Hospital episodes by gender for all 12 common psychiatric disorders, 1998/99-2019/20. 9

Supplementary Figure 4. Hospital episodes for psychiatric disorders by gender (male vs female and unspecified). Averaged across the full study period (1998/99-2019/2020). 10

Supplementary Figure 5. Hospital admissions (total counts) and median length of stay for all 12 common psychiatric disorders from 1998/99 to 2019/20. 11

Supplementary Figure 6. Trends in hospital admissions by psychiatric disorder as a percentage (%) of common psychiatric disorders, 1998/99-2019/20. 12

Supplementary Figure 7. Median length of stay in hospital by psychiatric disorder, 1998/99-2019/20. 13

Supplementary Figure 8. Trends in hospital episode rates for adults (15 years and older) by psychiatric disorder, 1998/99-2019/20. 14

Supplementary Figure 9. Trends in hospital episode rates for children (0-14 years old) by psychiatric disorder, 1998/99-2019/20. 15

Supplementary Table 1. ICD-10 codes for common mental, behavioural and neurodevelopmental disorders

Psychiatric disorder	ICD-10 code	Description
Dementia	F00	Dementia in Alzheimer's disease
	F01	Vascular dementia
	F02	Dementia in other diseases classified elsewhere
	F03	Unspecified dementia
Alcohol use disorder	F10	Mental and behavioural disorders due to use of alcohol
Substance use disorders	F11	Mental and behavioural disorders due to use of opioids
	F12	Mental and behavioural disorders due to use of cannabinoids
	F13	Mental and behavioural disorders due to use of sedatives or hypnotics
	F14	Mental and behavioural disorders due to use of cocaine
	F15	Mental and behavioural disorders due to use of other stimulants, including caffeine
	F16	Mental and behavioural disorders due to use of hallucinogens
	F17	Mental and behavioural disorders due to use of tobacco
	F18	Mental and behavioural disorders due to use of volatile solvents
	F19	Mental and behavioural disorders due to multiple drug use and use of other psychoactive substances
Schizophrenia	F20	Schizophrenia
	F21	Schizotypal disorder
	F22	Persistent delusional disorders
	F23	Acute and transient psychotic disorders
	F24	Induced delusional disorder
	F25	Schizoaffective disorders
	F28	Other nonorganic psychotic disorders
	F29	Unspecified nonorganic psychosis
Bipolar disorder	F31	Bipolar affective disorder
Depression	F32	Depressive episode
	F33	Recurrent depressive disorder
Anxiety	F40	Phobic anxiety disorders
	F41	Other anxiety disorders
OCD	F42	Obsessive-compulsive disorder
PTSD	F43	Reaction to severe stress, and adjustment disorders
Eating disorders	F50	Eating disorders
Personality disorders	F60	Specific personality disorders
	F61	Mixed and other personality disorders
Conduct disorders	F91	Conduct disorders

OCD = obsessive-compulsive disorder; PTSD = post-traumatic stress disorder

Supplementary Table 2. Three year moving averages for the total counts of hospital admissions by psychiatric disorder and population estimates, 1998/99-2019/20

	**1998/99 - 2000/01**	**2001/02 - 2003/04**	**2004/05 - 2006/07**	**2007/08 - 2009/10**	**2010/11 - 2012/13**	**2013/14 - 2015/16**	**2016/17 - 2019/20†**
**Common psychiatric disorders‡**	165992 (7913.68)	158216.33 (2968.78)	155471 (6778.57)	146210.33 (4012.01)	152656.67 (1271.62)	151281 (1921.48)	135984 (3005.09)
Alcohol use disorder	29104.67 (837.27)	29536.67 (1824.66)	39027 (2375.99)	44026.67 (3007.17)	46586 (1568.73)	45475.67 (1085.09)	41168 (1615.77)
Anxiety	5861.33 (132.49)	6101.33 (116.09)	6785.33 (170.12)	7952 (463.38)	8822 (109.66)	10026 (475.58)	11901.25 (1316.89)
Bipolar disorder	11739 (112.07)	12445.67 (666.69)	11773.67 (672.05)	10507.33 (325.73)	10536 (275.81)	10039 (356.32)	7713.25 (934.49)
Conduct disorders	685 (179.03)	378.67 (63.57)	257.67 (23.86)	281.33 (57.62)	237 (9.64)	228.67 (19.6)	208 (17.38)
Dementia	25411.33 (2116.28)	22392.33 (604.99)	19061.33 (2006.36)	14411.67 (717.84)	13232.33 (487.09)	11667.67 (485.54)	10283.75 (336.42)
Depression	34820 (2096.58)	31228.67 (1721.02)	25838.33 (2398.07)	21869.67 (584.78)	19960.33 (1184.44)	16630.33 (931.14)	13885.75 (354.69)
Eating disorders	1508.67 (67.26)	1514 (38.43)	1819 (147)	1935.67 (113.76)	2210.33 (219.29)	2896.67 (24.91)	3227.75 (265.57)
OCD	751 (71.25)	687.67 (26.1)	636.33 (25.15)	535.67 (32.87)	520.67 (16.77)	548.33 (25.97)	429.25 (44.78)
Personality disorders	8207.33 (622.5)	8030.33 (472.43)	7146 (801.63)	6953.33 (558.05)	9260 (566.86)	10534 (505.91)	9961.25 (553.92)
PTSD	6043.33 (335.51)	5658.33 (352.48)	4500 (622.03)	4396.67 (326.19)	5348 (290.53)	4960.67 (522.23)	4179 (134.38)
Schizophrenia	32910.33 (1215.23)	32206.67 (813.62)	30951 (2246.57)	27015 (865.03)	28914.33 (1507.11)	29452 (665.36)	24612.75 (2841.96)
Substance use disorders	8950 (720)	8036 (170.11)	7675.33 (572.93)	6325.33 (548.09)	7029.67 (189.53)	8822 (784.61)	8414 (278.07)
**Population estimates (per million)**	49.03 (0.17)	49.68 (0.2)	50.59 (0.32)	51.8 (0.34)	53.08 (0.35)	54.32 (0.38)	55.79 (0.39)

Standard deviations of the averages are included in parentheses. OCD = obsessive-compulsive disorder; PTSD = post-traumatic stress disorder; SD = standard deviation.). †Four year moving average to cover full study period. ‡Represents the summed total of the 12 most common psychiatric disorders (see Supplementary Table 1 for full diagnostic details).

Supplementary Table 3. Three year moving averages for the total counts of bed days by psychiatric disorder, 1998/99-2019/20

	1998/99 - 2000/01	2001/02 - 2003/04	2004/05 - 2006/07	2007/08 - 2009/10	2010/11 - 2012/13	2013/14 - 2015/16	2016/17 - 2019/20†
**Common psychiatric disorders‡**	5791734.65 (231953.31)	5961231.41 (356736.49)	6276071 (632980.33)	5867007.33 (569320.61)	6129239.67 (222762.22)	5469890.67 (351897.44)	4261584 (667254.52)
Alcohol use disorder	281263.51 (13985.11)	280761.7 (11507.64)	285078.33 (24113.6)	266700 (18792.78)	261207.33 (5898.11)	224329.33 (18112.21)	181217.75 (6765.92)
Anxiety	113050.1 (2250.3)	124489.32 (3022.75)	117542.33 (16139.97)	109328.67 (5222.64)	114959.33 (2936.86)	101976 (4264.72)	89382.25 (3509.29)
Bipolar disorder	549068.49 (7735.33)	621695.74 (46412.1)	662654 (42947.03)	578231.33 (25335.11)	587495 (14248.42)	529100.67 (51158.57)	399265.25 (58437.52)
Conduct disorders	11014.42 (2947.21)	8596.14 (1277)	4517.67 (211.07)	4314.33 (1138.98)	4988.67 (1983.27)	3386.67 (1267.15)	1451.25 (274.8)
Dementia	1131031.55 (66781.16)	1156618.98 (76976.03)	1111900.67 (129438.76)	920173 (27280.89)	825544.67 (45496.1)	614811.33 (75026.8)	432975.75 (74914.44)
Depression	1207040.09 (70787.44)	1145940.14 (26062.74)	1014750.67 (125795.17)	788952.67 (9124.85)	707700.67 (30831.39)	578330 (54335.85)	427544.5 (41195.96)
Eating disorders	64658.92 (5441.5)	65951.76 (7267.6)	90335.67 (4038.58)	91160 (10793.26)	105588 (14705.17)	126277.33 (3750.91)	118601 (11408.7)
OCD	30199.03 (2284.17)	30183.54 (2440.05)	31151 (2797.2)	24188 (2098.09)	31250.33 (1952.49)	30046.67 (4743.24)	23881 (2371.96)
Personality disorders	206726.03 (7636.65)	220211.74 (13114.18)	260421.67 (34961.19)	239464 (35122.29)	328876 (13169.28)	367050 (5216.08)	332458.75 (49186.31)
PTSD	98110.78 (4403.33)	99201.71 (2739.26)	86752 (10172.13)	82529.33 (14193.85)	92168 (2815.15)	87196.33 (7893.61)	73331.25 (3361.66)
Schizophrenia	1966549.5 (77643.67)	2076937.41 (204306.31)	2472708 (236136.46)	2655538.33 (454467.56)	2955122 (156645.17)	2689294.67 (147988.76)	2072472 (436563.84)
Substance use disorders	133022.24 (8633.54)	130643.22 (10464.38)	138259 (16446.21)	106427.67 (961.89)	114339.67 (10181.37)	118091.67 (5178.28)	109003.25 (10161.51)

Standard deviations of the averages are included in parentheses. OCD = obsessive-compulsive disorder; PTSD = post-traumatic stress disorder; SD = standard deviation.). †Four year moving average to cover full study period. ‡Represents the summed total of the 12 most common psychiatric disorders (see Supplementary Table 1 for full diagnostic details).

Supplementary Table 4. Three year moving averages for the median length of stay in hospital by psychiatric disorder, 1998/99-2019/20

	**1998/99 - 2000/01**	**2001/02 - 2003/04**	**2004/05 - 2006/07**	**2007/08 - 2009/10**	**2010/11 - 2012/13**	**2013/14 - 2015/16**	**2016/17 - 2019/20†**
**Common psychiatric disorders‡**	16.1 (0.33)	16.90 (0.27)	16.20 (0.49)	14.69 (0.45)	14.76 (0.49)	13.34 (0.06)	12.40 (0.94)
Alcohol use disorder	4.33 (0.58)	4.00 (0.00)	2.33 (0.58)	2.00 (0.00)	1.33 (0.58)	1.00 (0.00)	1.00 (0.00)
Anxiety	10.33 (0.29)	10.17 (0.58)	5.25 (2.61)	4 (0.87)	4.33 (0.58)	4.17 (0.58)	2.12 (1.65)
Bipolar disorder	32.33 (0.58)	33.67 (1.15)	33.67 (0.58)	29.00 (1.00)	29.00 (0.00)	29.33 (0.58)	28.5 (1.00)
Conduct disorders	3.33 (0.58)	3.33 (0.58)	2.00 (0.00)	1.33 (0.58)	1.67 (0.58)	1.00 (0.00)	1.00 (0.00)
Dementia	27.33 (1.91)	31.04 (1.8)	35.75 (6.11)	43.00 (2.18)	40.42 (4.23)	31.42 (0.8)	25.56 (5.54)
Depression	24.33 (0.29)	24.5 (0.50)	22.33 (1.26)	18.83 (0.58)	18.67 (0.29)	18.17 (0.76)	17.00 (1.47)
Eating disorders	19.00 (1.00)	20.00 (1.00)	16.67 (3.06)	15.00 (1.73)	14.00 (1.00)	12.33 (0.58)	9.75 (0.50)
OCD	20.67 (0.58)	23.00 (3.46)	21.83 (0.76)	15.33 (0.58)	21.00 (1.00)	20.00 (2.65)	21.00 (1.83)
Personality disorders	9.67 (0.29)	10.83 (1.53)	11.67 (1.28)	10.00 (1.50)	11.00 (0.00)	10.00 (0.50)	9.62 (0.63)
PTSD	8.00 (0.00)	9.00 (0.00)	8.00 (0.00)	7.33 (0.58)	7.33 (0.58)	7.67 (0.58)	7.50 (0.58)
Schizophrenia	27.5 (0.57)	26.15 (1.07)	28.71 (1.19)	25.62 (2.76)	24.46 (1.13)	22.33 (0.64)	22.88 (0.87)
Substance use disorders	6.37 (0.36)	7.15 (0.55)	6.2 (0.86)	4.78 (0.59)	3.85 (0.17)	2.70 (0.67)	2.83 (0.64)

Standard deviations of the averages are included in parentheses. OCD = obsessive-compulsive disorder; PTSD = post-traumatic stress disorder; SD = standard deviation.). †Four year moving average to cover full study period. ‡Represents the summed total of the 12 most common psychiatric disorders (see Supplementary Table 1 for full diagnostic details).

Supplementary Table 5. Trends in yearly hospital episode rates by age group (adults and children), 1998/99-2019/20

Psychiatric disorder	Best fitting model	Year of change†	AAPC (95% CI)	Estimated rates (per 1000 person-years)			Overall change from 1998/88 to 2019/20		Change from year of change to 2019/20	
				1998/99	Year of change	2019/20	Absolute change	Relative change	Absolute change	Relative change
**ADULTS (15y+)**										
Alcohol use disorder	Segmented	2010/11	3.44 (3.21-3.67)***	0.69	1.42	1.40	0.71	103.4%	-0.02	-1.4%
Anxiety	Segmented	2002/03	3.31 (3.07-3.55)***	0.16	0.16	0.32	0.16	98.1%	0.16	96.7%
Bipolar disorder	Segmented	2014/15	-1.93 (-2.19--1.66)***	0.33	0.31	0.22	-0.11	-33.6%	-0.09	-30.2%
Conduct disorders	Segmented	2002/03	-8.2 (-8.56--7.84)***	0.01	0.00	0.00	-0.01	-83.4%	0.00	-41.3%
Dementia	Segmented	2007/08	-2.18 (-2.41--1.95)***	0.79	0.56	0.50	-0.29	-37.0%	-0.06	-10.3%
Depression	Simple	..	-4.72 (-5.00--4.43)***	0.98	..	0.36	-0.63	-63.8%	..	..
Eating disorders	Segmented	2008/09	4.80 (4.6-5.00)***	0.03	0.04	0.09	0.06	167.6%	0.05	107.0%
OCD	Simple	..	-2.89 (-3.41--2.36)***	0.02	..	0.01	-0.01	-46.0%	..	..
Personality disorders	Segmented	2006/07	1.35 (0.73-1.97)***	0.24	0.19	0.31	0.08	32.4%	0.12	63.1%
PTSD	Simple	..	-1.65 (-2.44--0.86)***	0.15	..	0.11	-0.04	-29.5%	..	..
Schizophrenia	Simple	..	-0.41 (-0.90-0.08)	0.93	..	0.85	-0.08	-8.3%	..	..
Substance use disorders	Segmented	2008/09	0.07 (-0.18-0.32)	0.25	0.17	0.25	0.00	1.5%	0.08	47.4%
**CHILDREN (0-14y)**										
Alcohol use disorder	Segmented	2005/06	-7.60 (-8.04--7.15)***	0.19	0.25	0.04	-0.16	-81.0%	-0.21	-85.2%
Anxiety	Simple	..	6.61 (5.58-7.64)***	0.02	..	0.06	0.04	283.3%	..	..
Bipolar disorder	Segmented	2011/12	-3.09 (-5.69--0.43)*	0.00	0.00	0.00	0.00	-39.7%	0.00	-66.5%
Conduct disorders	Segmented	2005/06	-4.30 (-5.29--3.30)***	0.03	0.02	0.01	-0.02	-60.2%	0.00	-20.9%
Dementia	Simple	..	-16.15 (-21.87--10.01)***	0.00	..	0.00	0.00	-97.5%	..	..
Depression	Simple	..	5.58 (4.48-6.69)***	0.01	..	0.04	0.02	212.9%	..	..
Eating disorders	Simple	..	7.55 (6.76-8.35)***	0.02	..	0.10	0.08	361.2%	..	..
OCD	Simple	..	0.05 (-3.16-3.36)	0.00	..	0.00	0.00	1.0%	..	..
Personality disorders	Simple	..	4.93 (2.93-6.98)***	0.00	..	0.00	0.00	175%	..	..
PTSD	Segmented	2012/13	1.33 (-0.32-3.01)	0.00	0.01	0.01	0.00	33.1%	0.00	-43.7%
Schizophrenia	Simple	..	2.62 (1.54-3.71)***	0.01	..	0.01	0.01	72.2%	..	..
Substance use disorders	Simple	..	0.76 (-0.79-2.33)	0.01	..	0.01	0	17.2%	..	..

†Year of change is given only if the change-point (or segmented) model detected a significant deviation from a linear trend based on the pscore test. For change point models, AAPC represents the average annual percentage change from 1998/99-2019/20 while accounting for the uncertainty in the detected change points (Clegg et al., 2009; V. M. R. Muggeo, 2016). **p* < 0.05; ***p* < 0.01; ****p* < 0.001

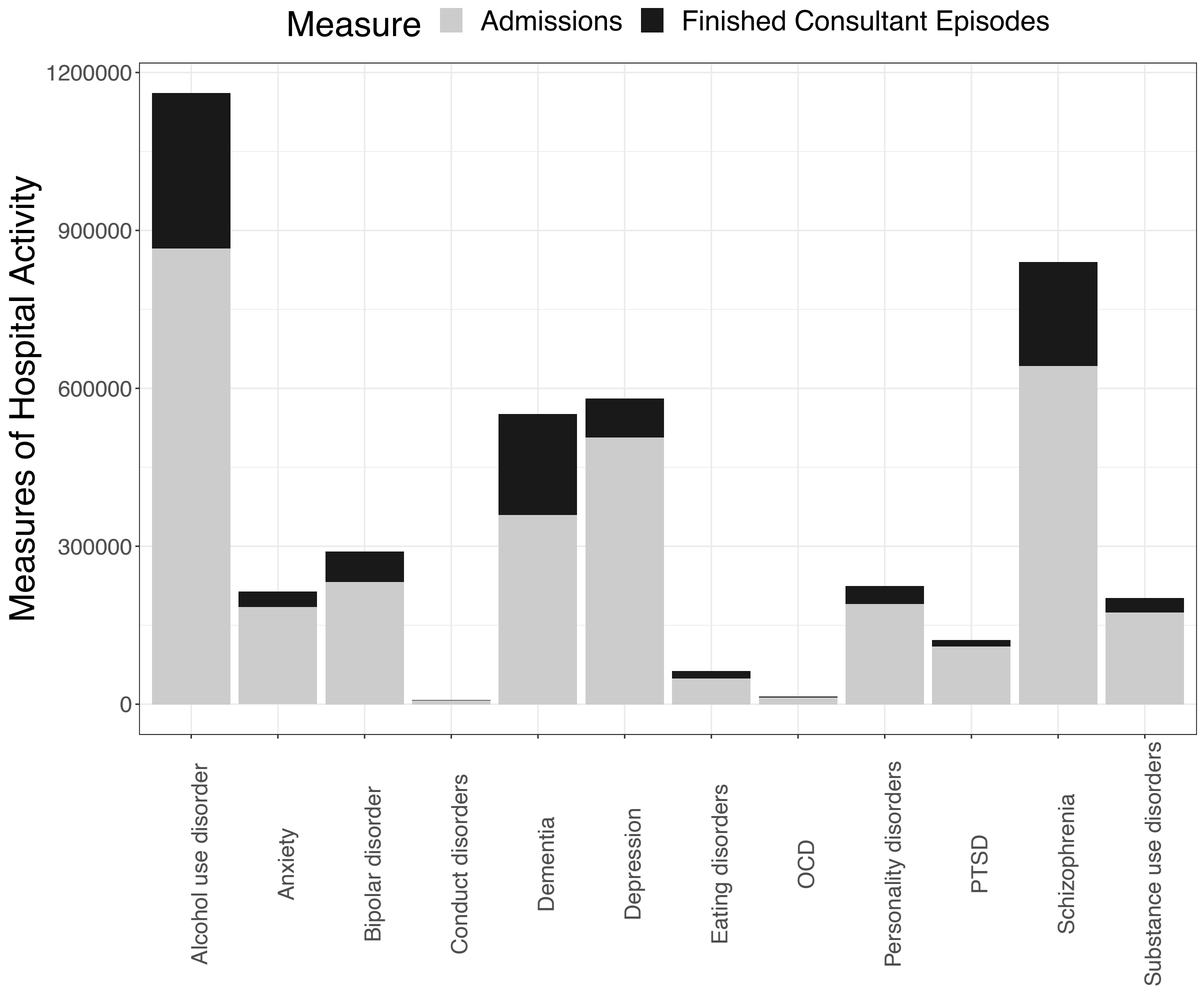

Supplementary Figure 1. Hospital activity for psychiatric disorders by measure (admission *vs* finished consultant episode). Averaged across the full study period (1998/99-2019/2020).

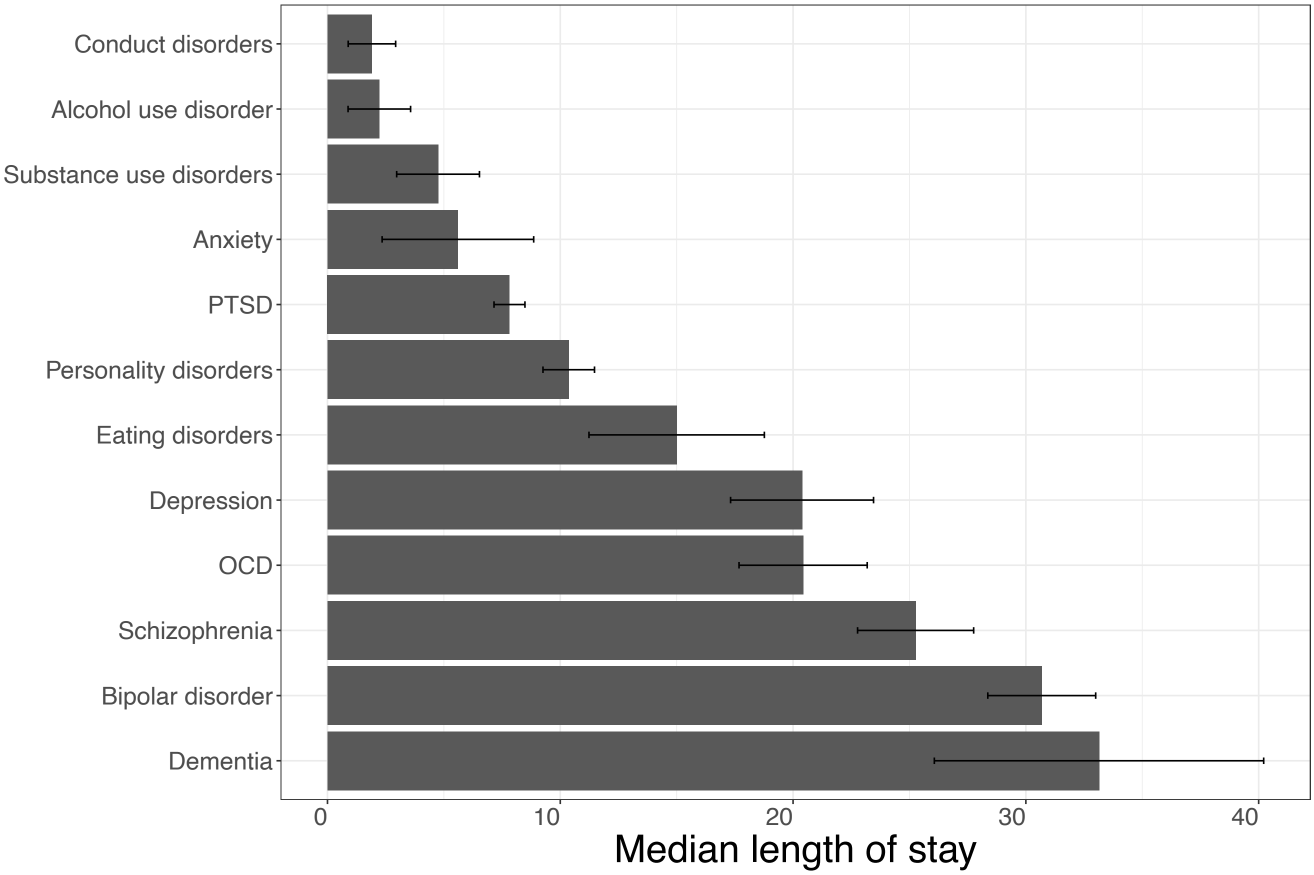

Supplementary Figure 2. Median length of hospital stay by psychiatric disorder. Averaged across the full study period (1998/99-2019/2020), with error bars representing standard deviations.

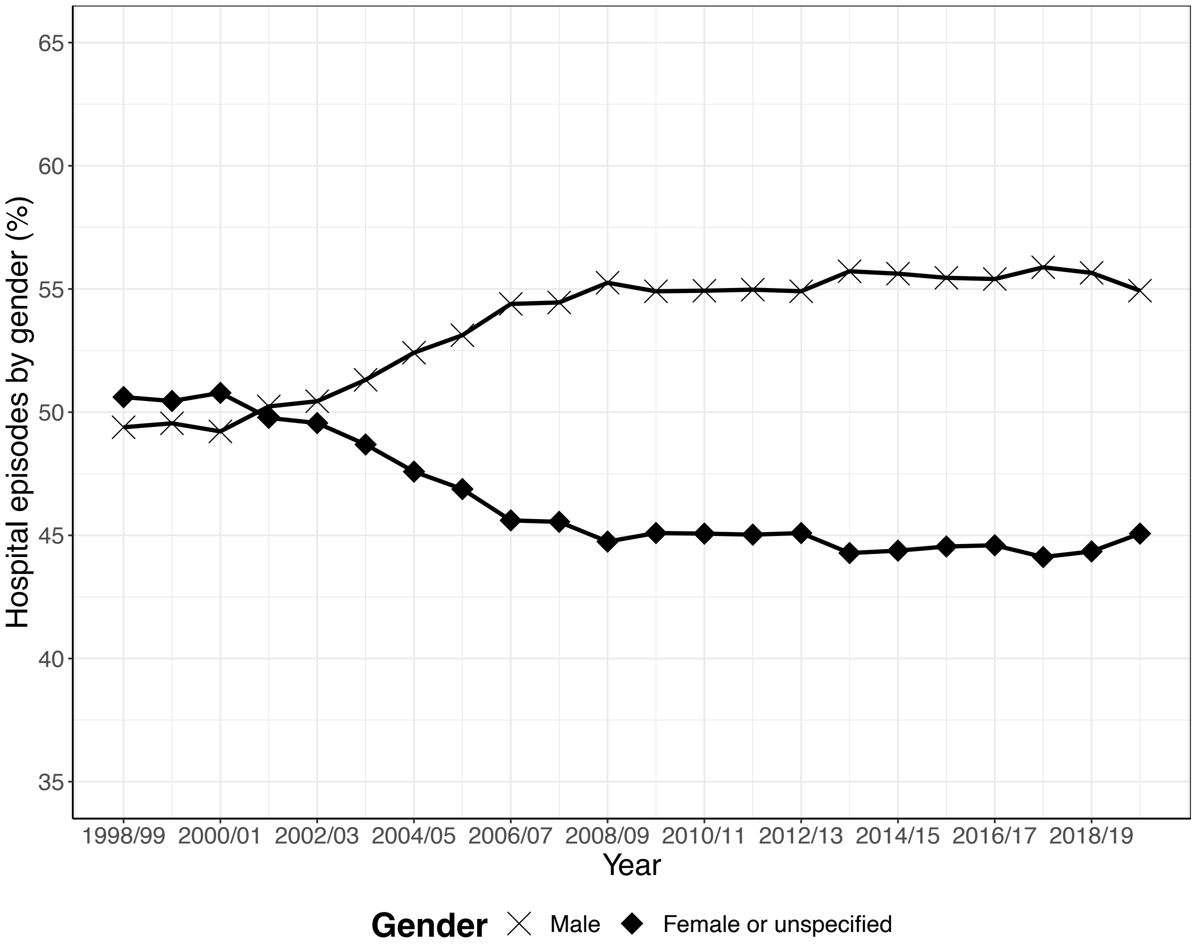

Supplementary Figure 3. Hospital episodes by gender for all 12 common psychiatric disorders, 1998/99-2019/20.

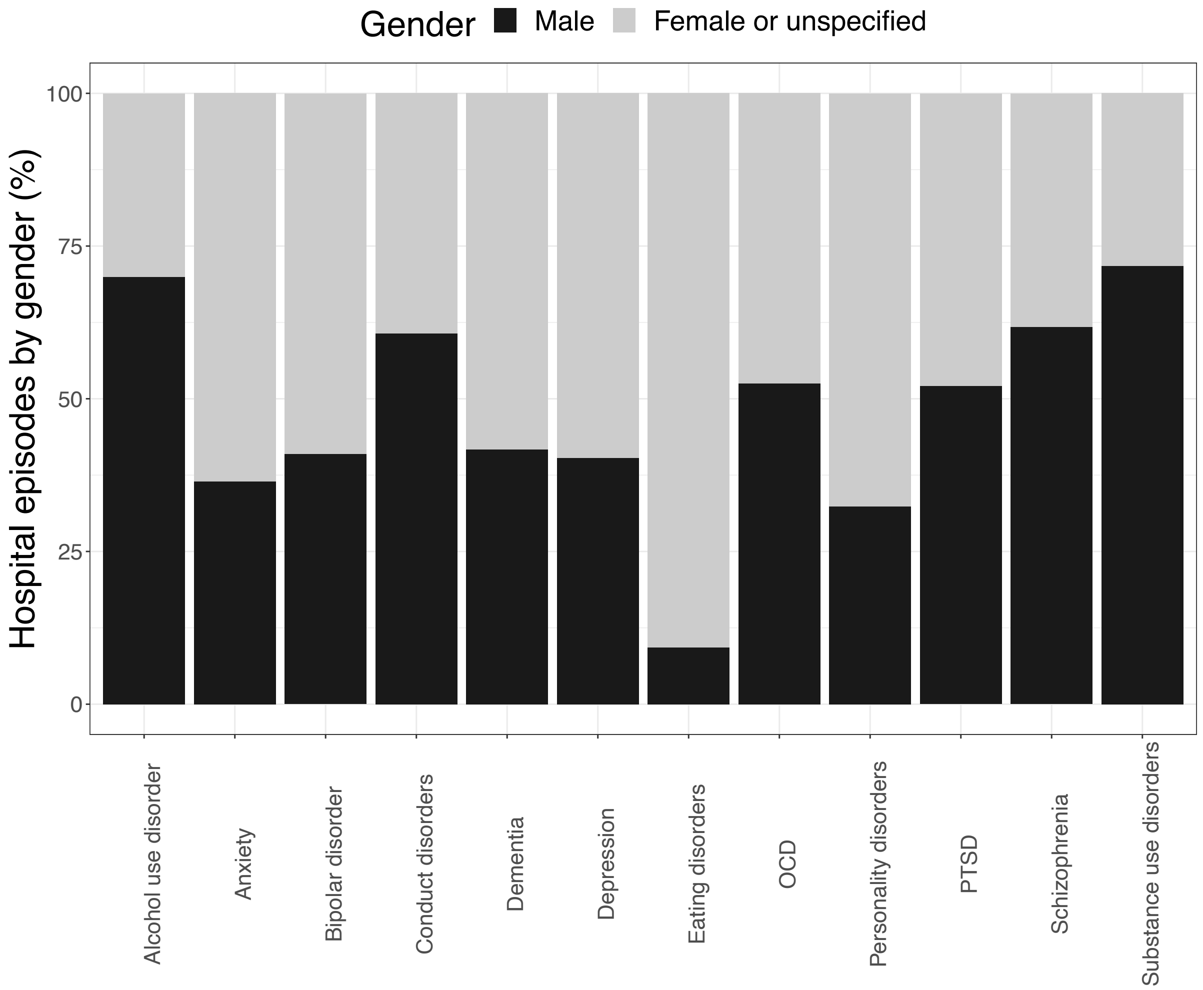

Supplementary Figure 4. Hospital episodes for psychiatric disorders by gender (male *vs* female and unspecified). Averaged across the full study period (1998/99-2019/2020).

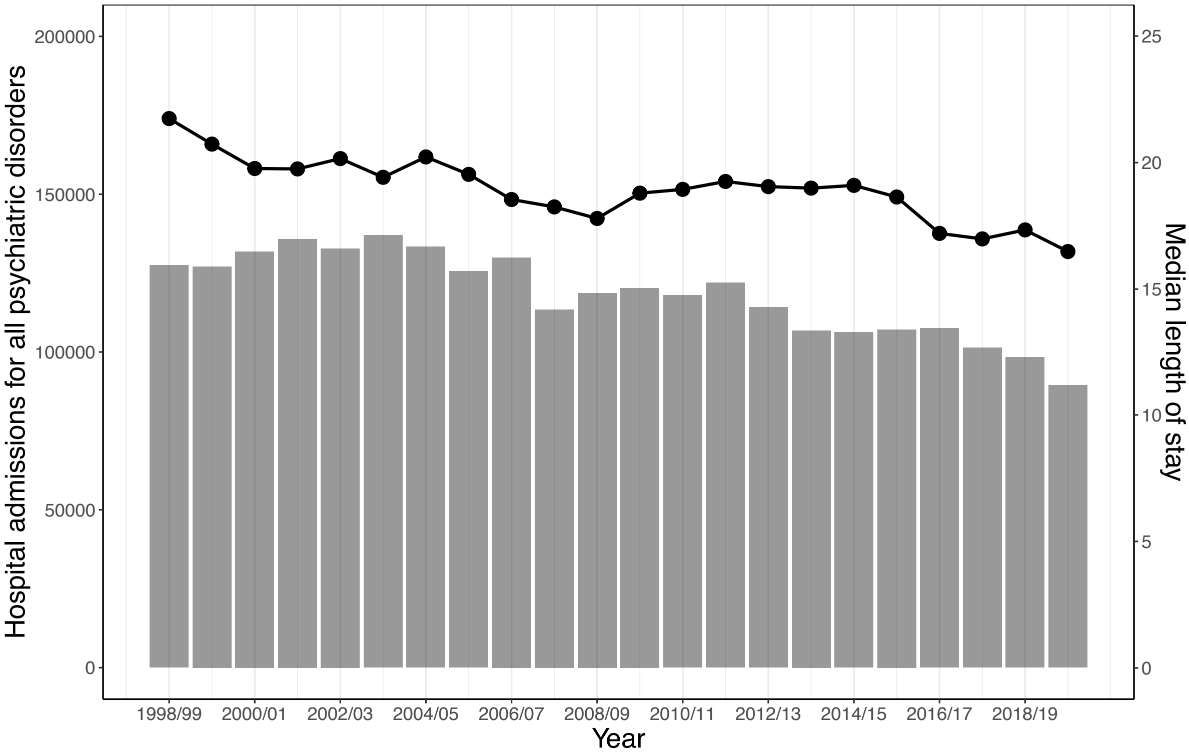

Supplementary Figure 5. Hospital admissions (total counts) and median length of stay for all 12 common psychiatric disorders from 1998/99 to 2019/20.

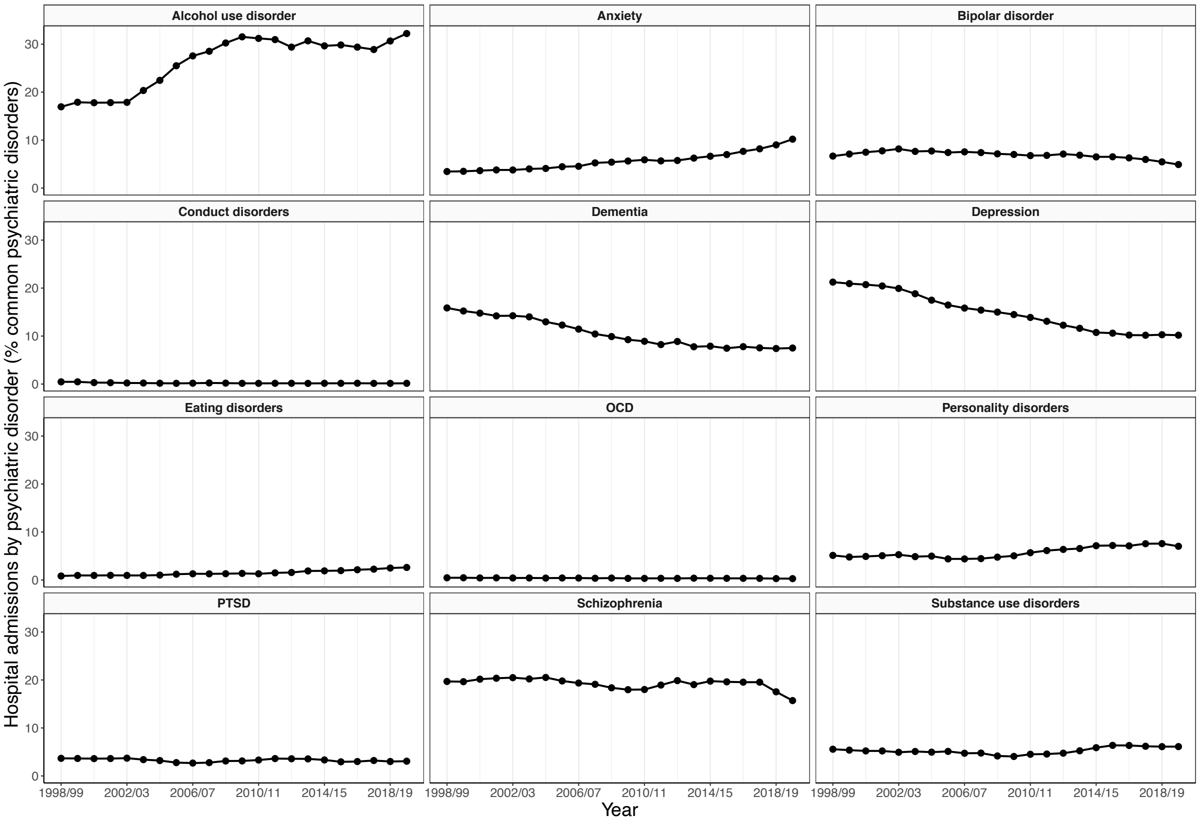

Supplementary Figure 6. Trends in hospital admissions by psychiatric disorder as a percentage (%) of common psychiatric disorders, 1998/99-2019/20.

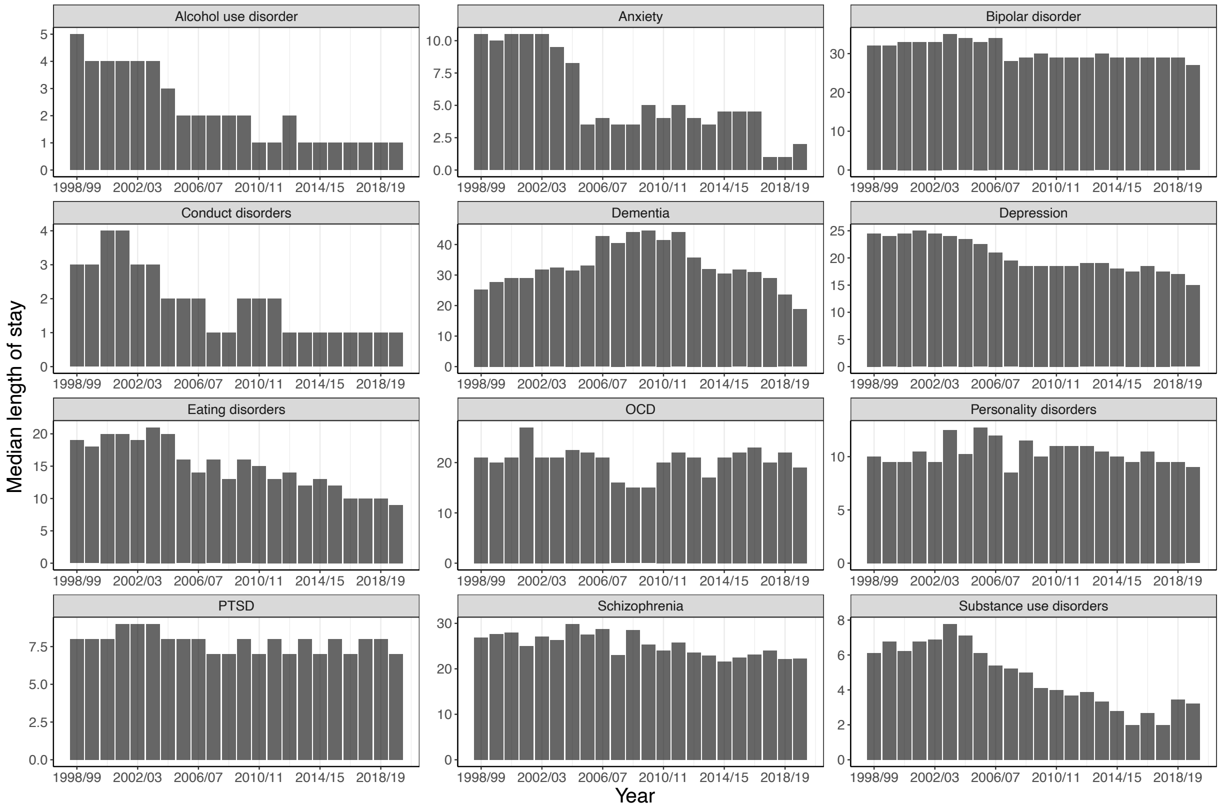

Supplementary Figure 7. Median length of stay in hospital by psychiatric disorder, 1998/99-2019/20.

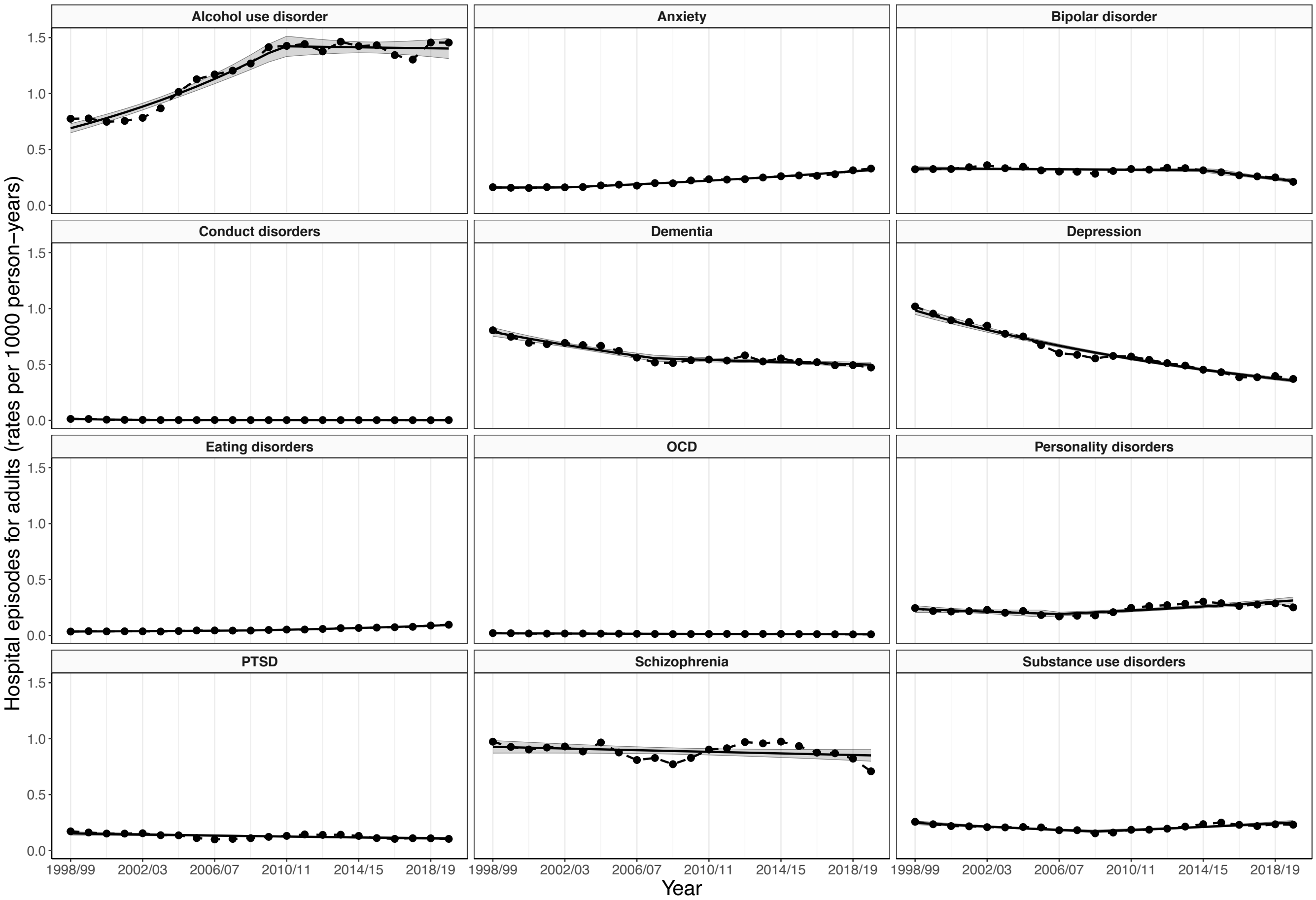

Supplementary Figure 8. Trends in hospital episode rates for adults (15 years and older) by psychiatric disorder, 1998/99-2019/20.

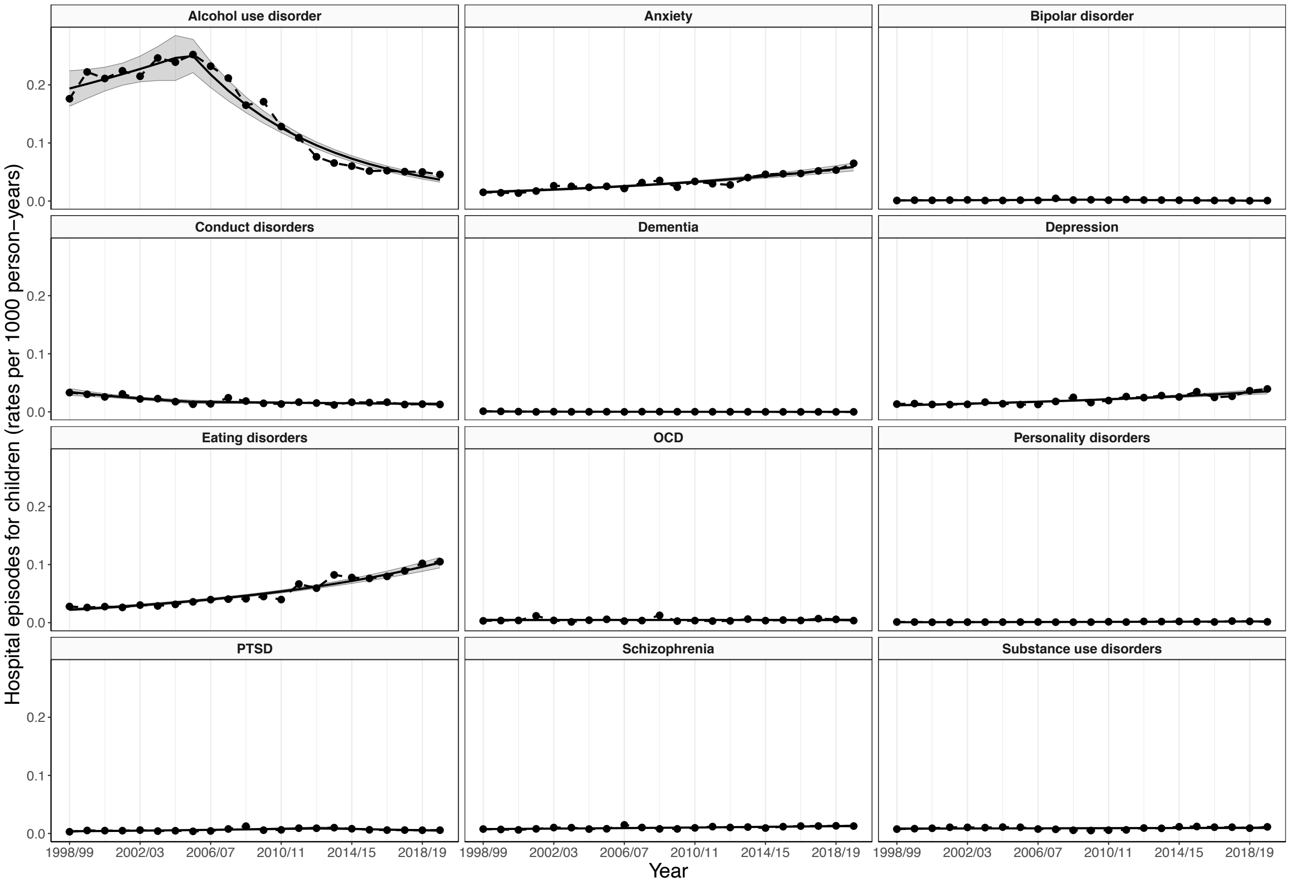

Supplementary Figure 9. Trends in hospital episode rates for children (0-14 years old) by psychiatric disorder, 1998/99-2019/20.
